# Evaluation of the Flexural Strength of Interim Restorative Materials in Fixed Prosthodontics

**Published:** 2016-09

**Authors:** Hanieh Mehrpour, Ehsan Farjood, Rashin Giti, Alireza Barfi Ghasrdashti, Hossein Heidari

**Affiliations:** 1 Biomaterials Research Center, Postgraduate Dept. of Prosthodontic Dentistry, School of Dentistry, Shiraz University of Medical Sciences, Shiraz, Iran.; 2 Dept. of Prosthodontics, School of Dentistry, Hormozgan University of Medical Sciences, Bandarabbas, Iran.

**Keywords:** Flexural Strength, Interim, Fixed Prosthesis

## Abstract

**Statement of the Problem:**

Mechanical properties of interim restorations are considered as important factors specially when selecting materials for long-term application or for patients with para-functional habits. Flexural strength is one of the most important components of these restorations.

**Purpose:**

The purpose of this study was to compare the flexural strength of five interim restorative materials.

**Materials and Method:**

Fifty identical samples sized 25×2×2-mm were made from five interim materials (TempSpan; Protemp 4, Unifast III, Trim, and Revotek LC) according to ADA specification #27. The specimens were stored in artificial saliva for 2 weeks and then thermocycled for 2500 cycles (5-55˚C). A standard three-point bending test was conducted on the specimens with a universal testing machine at a crosshead speed of 0.75mm/min. Data were analyzed by using one-way ANOVA and Tamhane’s post-hoc tests to measure the flexural strength of temporary materials.

**Results:**

One of the bis-acryl resins (TempSpan) showed the highest, and the light polymerized resin (Revotek LC) showed the lowest flexural strength. The mean values of flexural strength (MPa) for the examined materials were as follow: TempSpan=120.00, Protemp 4=113.00, Unifast III=64.20, Trim= 63.73 and Revotek LC=47.16. There were significant differences between all materials except Trim and Unifast III which did not show any statistical significant difference.

**Conclusion:**

Bis-acryl resins were statistically superior to traditional methacrylate and light-cured resins. Therefore, application of bis-acryl resins should be deliberated in patients with heavy occlusion and in cases that need long-term use of interim restorations.

## Introduction

One of the most important components of fixed prosthesis is temporary restorations.[1-2] During tooth preparation and before the insertion of final prosthesis, interim restorations must be delivered to patients. In prosthodontic treatment, the importance of provisional restorations is often ignored, resulting in problematic prosthesis which cannot protect the prepared teeth and supporting tissues sufficiently.[3-4]

The desirable restoration should provide essential mechanical, biological, and esthetic properties to become successful.[5] In order to achieve these purposes, some important characteristics including polymerization shrinkage, wear resistance, color stability, and strength of resin must be considered.[6-7]

From a mechanical viewpoint, the temporary restorative material should be chosen according to resistance to functional loads and removal forces.[5]

One of the important aspects of provisional restorations, especially in case of long-span interim prosthesis with short-height pontics and connectors, is their flexural strength.[5] The flexural strength of interim prosthesis also plays a critical role in patients with para-functional habits, bruxism, or clenching.[8] It causes much difficulty for both the patient and clinicians to keep the interim restorations intact. Any probable breakage of the prostheses leads to tooth movement as well as functional and esthetic problems. In addition, a repairing procedure may be boring and time consuming.[9]

Since the early days of temporary materials in 1930s, they have changed greatly from their first generation of acrylics and premade crown to more recent bis-acryl materials and computer-aided design/computer-aided manufacturing (CAD/CAM) restorations.[10]

In accordance to their composition, the interim restorative materials are categorized to 4 groups; polymethyl methacrylate, polyethyl or butyl methacrylate, microfilled bisphenol A-glycidyl dimethacrylate (Bis-GMA) composite resin, and urethane dimethacrylate (light-polymerizing resins).[5, 11]

Poly (methyl methacrylate) (PMMA) resins are relatively inexpensive; they render good color stability, good marginal accuracy, and excellent polishability. However, the main drawbacks of this type of resins are high polymerization shrinkage, exothermic polymerization, low strength, low wear resistance, and pulpal irritation as the result of excess free monomers. In comparison to PMMA resins, poly R′ methacrylates have low polymerization shrinkage and low exothermic reaction. However, they have limitations in clinical use such as low strength, low wear resistance, and low color stability. Bis-acryl composite resins are superior to methacrylate base resins as the result of their low polymerization shrinkage, low exothermic reaction, good wear resistance, and good strength. Nonetheless, they are expensive, brittle, less polishable, and much more difficult to repair.[12]

There is no interim material which can fulfill all requirements for every situation.[13-14] Therefore, clinicians always select their product based on the determinant factors such as cost effectiveness, esthetic, strength, marginal adaptability, and easy manipulation.[8]

As mentioned before, flexural strength is one of the significant characteristics of the interim fixed restorations which should be considered in multiple-unit or long-span prosthesis. Most previous studies have evaluated the flexural strength of interim prosthesis after polymerization. Limited studies have evaluated the effect of thermocycling on the interim material and its flexural strength.[15]

The purpose of this study was to evaluate the flexural strength of 5 interim restorative materials including TempSpan, Protemp 4, Unifast III, Trim and Revotek LC after thermocycling. The null hypothesis was that there are not significant differences in flexural strength of these temporary materials. 

## Materials and Method

Five interim restorative materials including Unifast III, Trim, Protemp 4, TempSpan, and Revotek LC were used in this study. They were indicative of the four types of interim materials described previously. Table 1 shows the characteristics of these materials.

**Table 1:** Temporary materials used in this study

**Product name**	**Manufacturer**	**Lot number**	**Composition**	**Polymerization**
Revote LC Unifast III Protemp 4 Trim Temp Span	GC corporation, Tokyo, Japan GC corporation, Tokyo, Japan 3M ESPE. AG, Seefeld, Germany Bosworth company, Skokie ,USA Pentron Clinical, orange CA,USA	1110121 1104081 452445 1007-323 4605909	Urethane dimethacrylate Methyl methacrylate Bis-acryl Vinyl ethyl methacrylate Bis-acryl	Light-cured Self-cured Self-cured Self-cured Dual-cured

A Plexiglas split mold was used to make specimens of 25×2×2-mm dimensions according to ADA specification #27.[16]

The interim materials were prepared according to the instructions of the manufacturing company; then they were injected to the mold. Trim and Unifast III were mixed manually; TempSpan and Protemp 4 were mixed automatically by using dispenser tip. Revotek LC was put into the mold by hand and a spatula.

A weight of 1.5 kg was fixed on the glass slab placed on the surface of the mold to remove the excess material from the mold, and to apply required pressure for complete polymerization. After the polymerization, samples were taken out of the mold and were precisely evaluated to detect any air bubbles. Problematic specimens were excluded from the study.

Finally, the samples were polished according to the manufacturers’ instructions.

For each material, 10 samples (50 in total) were made, and stored in artificial saliva at 37˚C for 2 weeks. They were then thermocycled for 2500 cycles between 5˚C and 55˚C. To measure the flexural strength of specimens, they were taken under the three-point bending test and placed on a universal testing machine with 0.75 mm/min crosshead speed.[8]

The fracture force was recorded in Newton and calculated in MPa by the following formula: 

S=3FL/2WH[2]

In this equation S=flexural strength, F=maximum fracture load, L=length of the specimen, W=width of the specimen, and H=height of the specimen.

Finally, data were analyzed by using one-way ANOVA and Tamhane’s post-hoc tests for multiple comparisons between and within groups. For all statistical analyses, the significance level of *α *= 0.05 was used.

## Results

Table 2 shows the mean and standard deviation of flexural strength for each examined material.

**Table 2:** Mean and standard deviation measures for flexural strength of interim materials

**Interim materials**	**Mean**	**Std.Deviation**	**95% Confidence Interval For Mean**	
			**Lower Band**	**Upper Band**
TempSpan Protemp 4 Unifast III Trim Revotek LC	120.2000 113.0000 64.2000 63.7300 40.1700	3.01109 5.61249 1.94993 1.72823 3.43448	118.0460 109.2147 62.8051 62.4937 37.7131	122.3540 116.7853 65.5949 64.9663 42.6269

The one-way ANOVA test showed a signiﬁcant difference between the flexural strength of 5 temporary materials (*p*< 0.05). The Tamhane’s post-hoc test which was carried out to compare each material with the four other groups indicated no significant difference in flexural strength of Trim and Unifast III specimens (*p*= 0.99). However, for other materials, significant differences were observed between the groups (*p*< 0.05). The highest flexural strength was seen in TempSpan (mean=120.00, max=124.00, and min=115.00), followed by Protemp 4 (mean=113.00, max=119.00, min=106.00). Trim (mean=63.70, max=66.00, min= 61.50) and Unifast III (mean=64.20, max=66.50, min= 61.20) showed lower flexural strength in comparison to bis-acryl resins. Finally, the lowest rank of flexural strength was noted in Revotek LC (mean=40.17, max= 44.20, min=34.80).

## Discussion

In this study the flexural strength of five interim resin materials including Unifast III, Trim, Protemp 4, TempSpan and Revotek LC were evaluated. Laboratory values of flexural strength under static loading may not reﬂect intraoral conditions; however, these values can help us to compare materials under controlled situations. They can also be considered as a useful predictor of clinical performance.[17]

In order to partially simulate oral environment, the specimens were stored in artiﬁcial saliva for 2 weeks and thermocycled for 2500 cycles between 5˚C and 55˚C. Then, all samples were examined by using the standard three-point bending test.

According to the results of this study, TempSpan and Protemp 4 bis-acryl composite resins had the highest ﬂexural strength. This result is similar to those of previous studies which proved that the ﬂexural strength of bis-acryl resins was higher than other conventional interim restorative materials.[9-10,17-20] Trim and Unifast III showed lower flexural strength than bis-acryl resins, and Revotek LC exhibited the lowest. Thus, the null hypothesis was rejected and significant differences were observed between flexural strength of the interim materials under examination.

Differences between ﬂexural strength of methacrylate base and bis-acryl resins is due to different monomer compositions. Multifunctional monomers of bis-acryl resins (such as Bis-GMA or TEGDMA) increase the strength of a resin as a result of cross-linking with other monomers.[18] Additional inorganic ﬁllers of these materials can also improve the strength and microhardness.[19-20] Besides all these virtues, the physical properties of composites and poly-acid modified composites may be enhanced by a protective layer over the surface of these restorative materials.[21]

On the other hand, conventional methacrylate resins are of low molecular weight, mono-functional, and have linear molecules which can reduce the strength and rigidity of restorations. Additionally, if they are not polymerized under pressure, their strength will decrease due to the trapped air bubbles.[9, 18, 22]

Poonacha *et al.*[23] compared the flexural strength and elastic modulus of three provisional materials. They concluded that the flexural strength of methacrylate resin reduced significantly; while, bis-acrylic composite resins showed a significant increase in its flexural strength after being stored in artificial saliva for 24 hours.

Balkenhol *et al.*[24] evaluated the flexural strength and flexural modulus of interim resin materials at different storage times. They showed the superiority of mechanical properties of resin-based composite materials over methacrylate resins; their result is in consistence with our study. They suggested the application of dual-curing interim resin materials in situations when high mechanical strength is needed. They also stated that in dual-curing materials such as TempSpan, the light curing initiation of the reaction results in a great amount of polymerization taking place at the beginning.

Yanikoğlu *et al.*[25] studied several interim restorative materials in different solutions and found that these solutions can not affect the fracture strength of these materials significantly. In consistence with our study, they concluded that composite based materials such as Protemp 4 showed the highest fracture strength and Takilon which was methacrylate-based exhibited the lowest.

Jo *et al.*,[12] in their evaluation of the flexural strength of interim resin materials, concluded that Protemp II has higher flexural strength and hardness in comparison with other autopolymerizing and light-curing resins.

Nejatidanesh *et al.*[8] evaluated the flexural strength of 7 interim materials. They found that TempSpan, which was composite-based, had the highest flexural strength; however, Trim showed the lowest flexural strength as it was methacrylate-based. We reached similar result in this study.

TempSpan, which showed the highest ﬂexural strength in the present study, is a dual-polymerizing material that has both auto- and light-polymerizing components which can increase the degree of polymerization; whereas, Protemp 4 is an autopolymerizing resin.[8]

Protemp is a bis-acryl resin with a flexible cross-linked polymer structure which improves the strength and hardness of the material.[20, 26] Since it is hydrophobic, it ensures minimal water uptake and, thus, reduces the plasticizing action.[20] The rigid central structure in bis-acryls decreases the dissolution of the resin-filler particles during their immersion in saliva.[27]

Kerby *et al.*,[28] in their evaluation of mechanical properties of urethane and bis-acryl interim resin materials, concluded that bis-acryl Protemp Plus showed significantly greater flexural strength and work-of-fracture than other resins after 24 hours of wet storage. They stated that the monomer system of Protemp Plus could produce polymers that were less susceptible to water sorption than urethane-based resins. This result is similar to our study which showed the superiority of bis-acryl resins over the light-cure resins.

Some authors claimed that during the bending test, Trim specimens were deﬂected without breakage, and the maximum force recorded by the universal testing machine was measured. Other researchers also found that after different storage conditions, extreme plastic deformation without fracture occurred and this resulted in the failure of Trim specimens.[14, 17, 24]

Sharma *et al.*[29] stated that the flexural strength of PMMA was comparatively better than the flexural strength of Urethane dimethacrylate (UDMA). Their result is consistent with our study. Moreover, they claimed that in order to remove the excess material during initial polymerization, UDMA specimens were taken out and then placed again in the mold for complete polymerization. This might deform the material and change its flexural strength.

One of the limitations of this study was the weak correlation between monotonic ﬂexural strength and resistance to fatigue loading. The fatigue tests proved to be more pertinent than monotonic ﬂexural strength. Therefore, testing materials under one consistent load may be inadequate to provide relevant information for long-term clinical performance.[30] Additionally, the interim resin materials can also be affected by saliva, food components, beverages and interactions among them in the oral environment.[20, 31-33]

There are no published studies to identify the appropriate mechanical properties of interim materials which can best aid the clinicians to predict the clinical performance of these materials *in vivo*.[34] Hence, all clinicians should be aware of various characteristics of restorations and select the suitable temporary material for each patient.[8]

## Conclusion

According to the present study, it can be concluded that bis-acryl interim materials present higher ﬂexural strength than methacrylate-based resins. Therefore, application of bis-acryls in patients with heavy occlusion can be considered. It seems that these interim restorations might work in long-term use. According to our results, we can also suggest the application of dual-curing temporary materials when high mechanical strength is needed. 
